# Understudied social influences on work-related and parental burnout: Social media-related emotions, comparisons, and the “do it all discrepancy”

**DOI:** 10.3389/fpsyg.2022.977782

**Published:** 2022-09-21

**Authors:** Kristen Jennings Black, Christopher J. L. Cunningham, Darria Long Gillespie, Kara D. Wyatt

**Affiliations:** ^1^Department of Psychology, University of Tennessee at Chattanooga, Chattanooga, TN, United States; ^2^Department of Emergency Medicine, University of Tennessee at Chattanooga, Chattanooga, TN, United States; ^3^Trueve Lab, Atlanta, GA, United States

**Keywords:** burnout, parental burnout, social comparisons, social media, social support, women, mothers

## Abstract

Recent societal changes, including a global pandemic, have exacerbated experiences of and attention to burnout related to work and parenting. In the present study, we investigated how several social forces can act as demands and resources to impact work-related and parental burnout. We tested two primary hypotheses in a sample of women who responded to an online survey (*N* for analyses ranged from 2376 to 3525). We found that social comparisons, social media use, negative emotions when comparing oneself to others on social media, and a high do it all discrepancy (feeling one should be able to do it all more so than perceptions that one can) were correlated with higher reports of work-related and parental burnout. Alternatively, positive emotions when comparing oneself to others and social support were related to lower reports of work-related and parental burnout. The influence of social media use on burnout was mediated by the emotions experienced when comparing oneself to others on social media. Tests of moderation indicated that social comparisons had stronger relationships with burnout for those with higher expectations that they should be able to do it all verses can do it all. Tests of social support as a moderator of the relationships between social demands and burnout were largely non-significant. Based on these findings, we make practical suggestions for interventions to increase positive emotions experienced from social media use, and to mediate the do it all discrepancy by redefining expectations around “doing it all.”

## Introduction

A global awareness of stress, mental health, and burnout has developed in recent years thanks in part to the COVID-19 pandemic and a barrage of societal, political, and economic changes (American Psychological Association, 2020, 2021; World Health Organization, 2022). The tenuous balancing acts that individuals and families were already managing to meet daily demands (e.g., American Psychological Association, 2015) turned into worst-case scenarios for many, threatening psychological and physical health. This phenomenon has been picked up and hyper-emphasized by media outlets, even to the point of being labeled “a burnout pandemic.”

Burnout is a serious strain state, typically studied in terms of exhaustion and disengagement as consequences of chronic overload in work settings (Demerouti et al., 2001; Kristensen et al., 2005). Although initially defined as experiences of emotional exhaustion, depersonalization, and a reduced sense of personal accomplishment for workers in “helping” professions (Maslach and Jackson, 1981), burnout is also experienced in other work (e.g., Ahola et al., 2006; Bakker et al., 2014) and non-work contexts (Mikolajczak and Roskam, 2018).

Most models of burnout focus on work-related antecedents, such as work-specific demands requiring effortful responses and resources that facilitate such responses. From the pertinent Job-Demands Resources model (JD-R; Demerouti et al., 2001) perspective, burnout is more likely to occur when work demands exceed available resources to meet demands (Bakker and Demerouti, 2017). Meta-analytic studies document consistent positive correlations of demands and negative correlations of resources to burnout (e.g., Crawford et al., 2010; Lesener et al., 2018).

Burnout is not limited to work experiences (Bianchi et al., 2014), but acting like it is limits our ability to understand and intervene to prevent severe cross-domain demands-resources imbalances. The Conservation of Resources Theory (COR; Hobfoll, 1989; Hobfoll et al., 2018) helps explain these complex personal demand-resource connections: Individuals desire to acquire, maintain, and protect their limited resources, which can include various states, objects, or energies. Stress and ultimately strain is experienced when our desired resources are threatened, depleted, insufficient, or absent. This holds true across all life domains, necessitating intentional resource allocation (Grawitch et al., 2010).

In the non-work, family role domain, *parental burnout* is increasingly being studied as a multidimensional construct involving exhaustion, feeling fed up or frustrated, emotional distancing, and reduced parental efficacy (Roskam et al., 2018). Parental burnout develops similarly to work-related burnout (i.e., from a chronic imbalance of demands and resources; Mikolajczak and Roskam, 2018; Roskam and Mikolajczak, 2021), but with more of a focus on one’s personal and family resources. There is evidence that work-related and parental burnout are related yet distinct phenomena with differing antecedents and outcomes (Mikolajczak et al., 2020).

Given changing sociocultural dynamics, particularly pertaining to the increasing evidence of good and bad effects of social media use (e.g., Baccarella et al., 2018) and increasing isolation and loneliness (even pre-COVID; e.g., Holt-Lunstad, 2017), we designed this study to examine the effects of several social factors on work and parental burnout. These social factors are particularly important, given the complex effects our social relationships and influences can have (as demands and resources) on health and wellbeing. In particular, social comparisons that involve general tendencies to compare ones’ abilities and opinions to others’ (e.g., Festinger, 1954), can serve a positive purpose in gaining self-awareness or self-improvement. However, greater tendencies or social pressures to engage in social comparisons are often themselves demands which can harm one’s self-worth or mental health (e.g., Butzer and Kuiper, 2006; Wemken et al., 2021). These social/cognitive demands can directly require effort from us (e.g., refocus attention, reframe thoughts, or change actions to align with comparative others) or act as demands by threatening valuable resources, such as self-worth or positive emotional states (Muller and Fayant, 2010; Johnson, 2012).

Opportunities for social comparisons have grown substantially with the widespread adoption of social media. Social media use can have both positive connective benefits (e.g., Sormunen et al., 2020; Chen and Xu, 2021) and negative comparison-related or expectation-related effects on users’ wellbeing (e.g., Feinstein et al., 2013; Nisar et al., 2019). For women with children, social media groups have been found to be helpful sources of support but can evoke a wide range of emotional experiences and pressures (César et al., 2018). In the present study, we considered how social comparisons in general are linked as demands to work-related and parental burnout. We also considered the effects of time spent on social media as a demand, mediated by emotions associated with comparisons when using social media (Park and Baek, 2018). We also examined socially derived expectations regarding “doing it all,” a message that impacts many women and is commonly depicted in popular media but is not often studied. We expect that, similar to dispositions toward high personal standards (e.g., perfectionism; Sorkkila and Aunola, 2020), a high “do it all discrepancy” may contribute to burnout.

From a resource perspective, social support and the availability of high-quality social connections generally benefit individuals’ health and wellbeing (e.g., Taylor, 2020; Gable and Bedrov, 2022). The protective role of social support has been incorporated into numerous stress-related theories, such as the demands-control-support model (Van Der Doef and Maes, 1999), which describes how social support may help to counter negative effects of work demands on health and wellbeing. There is consistent evidence that social support at work is related to fewer burnout symptoms (Velando-Soriano et al., 2020). Further, availability and satisfaction with social support have been correlated with less parental burnout (Ardic, 2020; Szczygiel et al., 2020).

**FIGURE 1:**
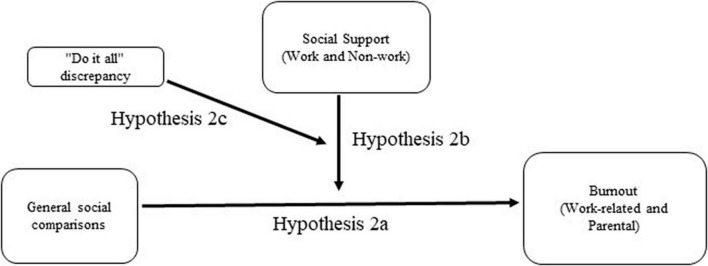
Depiction of Hypothesis 1, where social media relates to work-related and parental burnout *via* emotions, moderated by social support.

**FIGURE 2:**
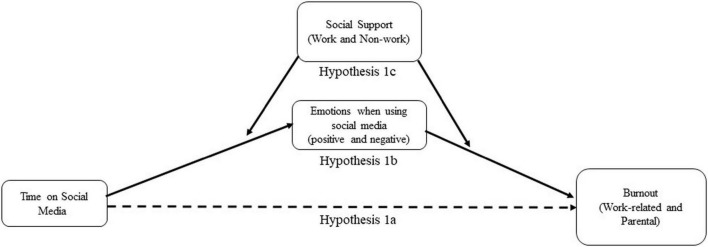
Depiction of Hypothesis 2, where social comparisons interact with social support and do it all discrepancy to predict work-related and parental burnout.

It is likely that social support from work and non-work sources may function as a resource to help in responding to social demands as well, though there are certainly complexities in the effectiveness of support (i.e., provided in the right manner, at the right time; Beehr et al., 2003; Gray et al., 2019). In the present study, we considered social support from work and family roles as both a main effect and moderator of the relationships between other social demands and burnout. Although support as a resource should be protective against burnout, beneficial effects may be limited if not matched to the domain in which demands are being most strongly experienced (e.g., work support affecting parental burnout or non-work support impacting work burnout; Beehr et al., 2010). To summarize, we proposed two hypotheses (Figures 1, 2).

*Hypothesis 1*: (a) Social media use is positively associated with work-related and parental burnout, (b) this relationship is mediated by emotions experienced when using social media, and (c) this indirect effect is moderated by perceived work and non-work social support, such that the direct and indirect relationships are weaker when social support is high.

*Hypothesis 2*: (a) A tendency to compare oneself to others is positively associated with work-related and parental burnout, (b) this relationship is moderated by perceived work and non-work social support, such that the relationship is weaker when support is high; and (c) this moderation effect is further moderated by the extent to which an individual perceives she should be doing more than she can (“do it all discrepancy”).

## Method

### Participants and procedure

Data were gathered from October through December 2021 *via* internet-based survey as part of a larger study of women, most of whom manage multiple life roles (e.g., mother, spouse, employee). Participants were recruited in partnership with multiple consumer products and services organizations who shared the survey with their user/member lists by email and various social media channels. All research methods were reviewed by the university’s institutional review board (IRB; Federal-wide Assurance ID: FWA00004149) and exempted from IRB oversight given the carefully designed data collection process. Participants (and non-participants) had the opportunity to enter an incentive drawing for one of several gifts (e.g., Amazon.com gift cards, The Company Store merchandise) upon study completion. After careful review of raw data and removal of respondents who failed one or more attention checks, failed an integrated bot-detection protocol, and/or did not respond to at least 15% of the survey, the final sample included 4,205 participants. Because of missing data, sample sizes for hypothesis tests ranged from 2,376 to 3,525 (precise *N*-values are reported with results summary tables).

Despite multiple efforts to reach a racially and ethnically diverse sample, 95.6% of respondents were non-Hispanic/Latinx and 91.2% were white. The average age of respondents was 42.53 years (SD = 10.84). Most respondents had completed at least a Bachelor’s degree (84%) and approximately 88% of this sample was married or living with a partner. Most (76%) had at least one dependent under age 18. Most respondents were involved in full-time work (65.5%), with fewer in part-time work (18.4%) or not working (16.2%). The average reported household income range for these participants was $100,000 to $149,000, with roughly 80% reporting a household income of $75,000 or more. We acknowledge this demographic profile represents a well-resourced group; any observed effects could be expected to be stronger in less-resourced groups.

### Measures

Our hypotheses were tested with data gathered using the following measures. All multi-item scales demonstrated adequate internal consistency reliability (see Table 1 for Cronbach’s alpha values).

**TABLE 1:** Descriptive statistics and bivariate correlations for all study variables.

	*M*	SD	1.	2.	3.	4.	5.	6.	7.	8.	9.	10.	11.	12.	13.	14.	15.	16.	17.
1. Age	42.09	10.40	–																
2. Education	4.91	1.39	–0.21*	–															
3. Marital status	0.11	0.32	0.21*	–0.14*	–														
4. Number of dependents	1.52	1.08	–0.33*	0.04*	–0.29*	–													
5. Work hours	2.50	0.75	–0.25*	0.26*	0.02	–0.02	–												
6. Volunteer hours	1.69	1.19	0.19*	–0.04*	0.03*	0.03	–0.15*	–											
7. Home overload	4.36	0.88	–0.24*	0.10*	–0.15*	0.21*	0.17*	–0.07*	–										
8. Work overload	3.93	1.13	–0.09*	0.15*	–0.05*	0.07*	0.16*	–0.04	0.39*	–									
9. SM hours	6.61	6.36	–0.09*	–0.07*	0.01	0.03	–0.04*	–0.01	0.01	–0.02	–								
10. Positive SM emotions	2.82	0.82	0.01	–0.01	–0.03	0.03	0.04*	0.04*	–0.02	0.01	0.15*	(0.46)							
11. Negative SM emotions	3.12	0.92	–0.28*	0.08*	–0.09*	0.13*	0.05*	–0.07*	0.20*	0.12*	0.11*	–0.06*	(0.41)						
12. Social comparisons	3.35	0.85	–0.22*	0.06*	–0.08*	0.10*	0.01	–0.04*	0.16*	0.12*	0.13*	–0.01	0.53*	(0.79)					
13. Do it all discrepancy	1.69	1.42	–0.22*	0.09*	–0.10*	0.11*	0.05*	–0.05*	0.30*	0.20*	0.06*	–0.14*	0.28*	0.29*	–				
14. Work support	3.62	0.94	–0.08*	0.09*	–0.08*	0.05*	0.16*	–0.01	–0.05*	–0.07*	–0.03	0.11*	–0.04*	–0.06*	–0.07*	(0.55)			
15. Non-work support	3.01	0.69	–0.03	0.14*	–0.09*	–0.06*	0.09*	0.00	–0.12*	–0.08*	–0.04*	0.10*	–0.13*	–0.12*	–0.13*	0.31*	–		
16. Work-Related burnout	3.22	0.67	–0.26*	0.04*	–0.03	0.16*	0.09*	–0.09*	0.38*	0.37*	0.08*	–0.12*	0.28*	0.27*	0.35*	–0.25*	–0.26*	(0.87)	
17. Parental burnout	1.79	1.36	–0.27*	0.02	–0.08*	0.20*	–0.11*	–0.05*	0.26*	0.14*	0.09*	–0.12*	0.28*	0.25*	0.32*	–0.10**	–0.29*	0.54*	(0.99)

*p < 0.05. N range = 2590–3525. Along the diagonal, Cronbach’s alpha is displayed for multi-item scales; for two-item scales, the correlation between items is displayed. SM, social media. Education coded as highest degree earned, higher values represent a more advanced degree. Martial status: 0 = married or committed relationship, 1 = single, widowed, or divorced.

*Social media use* was operationalized in terms of hours per week spent using social media. For additional context, we also asked participants how often they post, contribute, or interact with others *via* social media (1 = never or almost never to 6 = multiple times per day).

*Emotions associated with social media comparisons* were measured with four items adapted from Park and Baek (2018) for broader applicability to all social media platforms and for easier responding. Participants were asked: “How likely are you to feel each of the following emotions when you compare yourself to others on social media (e.g., Facebook, Instagram, TikTok, Twitter, etc.)?,” with four emotional item prompts rated on a scale from 1 = very unlikely to 5 = very likely. Positive (optimism, pride) and negative (envy, worry) emotions were averaged, with higher scores indicating stronger negative or positive emotions while using social media.

*Do it all expectation discrepancy* was operationalized with participants responses (on a scale from 1 = not at all to 5 = completely) to two items: “To what extent do you feel you should be able to ‘do it all’ as a parent?,” and “To what extent do you feel that you can ‘do it all’ as well as you would like to?,” Subtracting the second response from the first yielded a do it all discrepancy score. Negative values resulted when can exceeded should, values near zero resulted when can and should were similar, and positive values resulted when can was less than should.

The extent to which participants engaged in *social comparisons* was measured with a subset of four items representative of the performance/achievement comparisons subscale from Gibbons and Buunk (1999). We changed the item referent to be more applicable to our respondents (e.g., “I always pay a lot of attention to how I do things compared with how others do things.”). Higher scores (rated on a scale of 1 = disagree strongly to 5 = agree strongly) indicated a stronger tendency to compare oneself to others.

*Social support* was measured with three items adapted from the NIOSH (2021) well-being questionnaire. Non-work support was measured with one item (“How often do you get the social and emotional support you need from friends, family, or others outside of work?,” rated on a scale of 1 = never to 4 = always), while work support was measured with two items assessing perceived supervisor and coworker support when needed (rated on a scale of 1 = disagree strongly to 5 = agree strongly).

*Work-related burnout* was measured with the 13-item Copenhagen Burnout Inventory (Kristensen et al., 2005), which primarily focuses on personal and work-related exhaustion. *Parental burnout* was measured with the 23-item Parental Burnout Assessment (Roskam et al., 2018), which evaluates the frequency with which parents perceive multiple aspects of burnout in this domain linked to exhaustion, parental self-perceptions, frustrations, and emotional distancing. Higher scores on both measures indicated higher levels of either form of burnout.

*Covariates* that could affect burnout were accounted for in our analyses. These included self-reported age, education (measured as highest degree earned), marital status (dummy coded as in a committed relationship or single/widowed/divorced), number of dependents (asked as total number of children under 18), hours worked per week, frequency of volunteering (to account for work-related obligations of those not formally employed), and self-reported time overload in the work and home domain (i.e., I never seem to have enough time to get everything done at home; I never seem to have enough time to get everything done on my job; response options 1 = disagree strongly to 5 = agree strongly). Other demographic variables (e.g., race, ethnicity) were assessed but were not included as covariates in our models because they did not exhibit strong relationships with other study variables.

### Statistical analyses

All analyses were conducted in SPSS version 27. Descriptive statistics were computed for all study variables to ensure normality and to explore frequencies of responses to nominal and ordinal variables. Pearson’s correlation coefficients were calculated for all continuous variables and dummy-coded nominal variables. Significance tests to compare the strength of selected correlations were completed with the cocor program (Diedenhofen and Musch, 2015). Tests of moderation and mediation were done with Hayes (2022) PROCESS macro version 4.0 for SPSS, using 5000 bootstrapping iterations to generate robust 95% confidence intervals that we used to determine statistical significance. In these models, we controlled for the covariates previously mentioned.

## Results

From the descriptive statistics (Table 1), participants spent an average of 6.6 hours on social media each week; in a follow-up item, 68% of women reported posting to social media at least once a week. When comparing themselves to others on social media, women were more likely to report feeling negative than positive emotions (e.g., 53% likely or very likely to experience envy, 36% likely/very likely to experience worry vs. 26% likely/very likely to feel optimism, 24% likely/very likely to experience pride). Notably, these emotions were correlated with work-related and parental burnout at a significantly higher magnitude compared to time using social media. In addition, negative emotions were significantly more strongly correlated with burnout than positive emotions.

Participants reported moderate levels of social comparisons and held high perceived expectations around managing different life roles. Around 64% of women very much or completely felt they *should* be able to do it all while only 7% felt they very much or completely *could* do it all. Only 6% of the sample reported a negative do it all discrepancy (should < can); 15% reported zero discrepancy while 78% reported positive discrepancy (should > can). Both social comparisons and a higher do it all discrepancy were positively related to work-related and parental burnout. Regarding support, both work and non-work support correlated negatively with burnout. It is also interesting to note that work-related and parental burnout were moderately correlated; however, the average level of reported work-related burnout was higher than parental, despite similar instructions and response scales for both measures.

We tested Hypothesis 1 with four separate PROCESS models (i.e., negative or positive emotions as mediators with work-related or parental burnout as outcomes). The full results are summarized in Supplementary Table 1. First, considering the direct effects of social media use (Hypothesis 1a), more time spent on social media was significantly and positively linked to negative and positive emotions associated with social media-based social comparisons. As was the case in the bivariate correlations, social media use was also significantly related to work-related burnout and parental burnout above and beyond covariates in all models.

Second, the hypothesized indirect effects (Hypothesis 1b) were statistically significant; social media use was related to more negative emotions when comparing oneself to others on social media, which were then positively related to work-related burnout (Model 1.1) and parental burnout (Model 1.2). There were some nuances in Model 1.1 examining work-related burnout as the outcome, where this indirect effect was non-significant when non-work support was high, but work support was low. In Model 1.2 with parental burnout, the indirect effect of negative emotions became noticeably weaker and non-significant when non-work support was high, regardless of the level of work support. These findings, however, did not indicate clear or consistent moderation by social support.

Social media use was also related to more positive emotions when comparing oneself to others on social media, and these positive emotions were negatively related to work-related burnout (Model 1.3). Indirect paths were significant at all levels of work and non-work support in Model 1.3. There was also an indirect effect *via* positive emotions on parental burnout in Model 1.4, with significant indirect effects for all but one combination of social support, which again was not strong evidence for a significant moderated effect.

Third, regarding the hypothesized moderating effects of social support (Hypothesis 1c), no significant interactions were identified across Models 1.1 through 1.4 – social support did not moderate the mediated relationships just discussed. In terms of main effects, both work and non-work support were significantly associated with work-related burnout (Models 1.1 and 1.3) and non-work support was associated with parental burnout (Models 1.2 and 1.4). Only non-work support was related to fewer negative emotions when using social media (Models 1.1 and 1.2). Both work and non-work support were related to more positive emotions (Models 1.3 and 1.4).

We similarly tested Hypothesis 2 with four separate PROCESS models (work or non-work support as moderator and work-related or parental burnout as outcome), summarized fully in Supplementary Table 2. General social comparisons were positively related to work-related and parental burnout in all models, supporting Hypothesis 2a. Across these analyses, a greater positive do it all discrepancy (should > can) was also significantly and positively associated with work-related and parental burnout. Work and non-work support were negatively related to work-related and parental burnout.

Regarding the hypothesized interaction effects, the relationship between social comparisons and parental burnout was moderated by do it all discrepancy (Models 2.2 and 2.4), such that the relationship became stronger as the do it all discrepancy became more positive (Supplementary Figure 1). Thus, women were more likely to report burnout as a result of social comparisons, when the gap was bigger between feeling they should versus can do it all. This interaction was significant in both the work and non-work support models, though two- and three-way interactions involving support were all non-significant.

When predicting work-related burnout (Models 2.1 and 2.3), all two-way interactions involving non-work support were non-significant. However, a significant three-way interaction was observed involving social comparisons, work support, and do it all discrepancy (Supplementary Figure 2). Specifically, the interaction between general social comparisons and work support was only significant at lower, but still positive, do it all discrepancy levels. This suggests that work support may only be effective, or at least is more noticeable in buffering the negative effects of social comparisons, when an individual’s can-do feelings are closer to their should do expectations. In sum, Hypothesis 2a was not supported, but evidence of one three-way interaction and some two-way interactions involving do it all discrepancy, provided partial support for Hypothesis 2b. A summary of the supported hypotheses overall is provided in Table 2.

**TABLE 2:** Summary of results of hypothesis tests.

*Hypothesis 1*	Model 1.1	Model 1.2	Model 1.3	Model 1.4
Outcome:	Work Burnout	Parental Burnout	Work Burnout	Parental Burnout
Mediator:	Negative Emotions	Negative Emotions	Positive Emotions	Positive Emotions
(a) Social media use is positively associated with burnout	Yes	Yes	Yes	Yes
(b) Emotions mediate the social media-burnout relationship	Yes	Yes	Yes	Yes
(c) Direct/Indirect paths moderated by social support (work and non-work)	No	No	No	No

***Hypothesis 2***	**Model 2.1**	**Model 2.**	**Model 2.3**	**Model 2.4**

Outcome:	Work Burnout	Parental Burnout	Work Burnout	Parental Burnout
Moderator:	Non-work Support	Non-work Support	Work Support	Work Support
(a) Social comparisons are positively associated with burnout	Yes	Yes	Yes	Yes
(b) The social comparisons-burnout relationship is moderated by social support	No	No	No	No
(c) The moderated effect of social support is further moderated by the do it all discrepancy (three-way interaction)	No	No *Significant two-way interaction between social comparisons and do it all discrepancy	Yes	No *Significant two-way interaction between social comparisons and do it all discrepancy

Yes = a significant effect was found (p < 0.05, confidence interval did not contain zero). No = a significant effect was not found. *Noteworthy finding not explicitly stated in hypotheses.

## Discussion

The COVID-19 pandemic and other recent societal changes have exacerbated already high demands and reduced chronically low resources for many people, especially women. Social relationships are generally seen and experienced as resources (e.g., Taylor, 2020; Gable and Bedrov, 2022), but our increasing use of social media and ongoing social trends toward isolation can be strain-inducing (e.g., Feinstein et al., 2013; Nisar et al., 2019), particularly for women with children (e.g., César et al., 2018). We examined how social forces can act as both demands and resources in relation to work-related and parental burnout. We found that social media use can trigger both positive and negative emotions; positive emotions (optimism, pride) were related to lower work-related and parental burnout, but negative emotions (envy, worry) were related to higher burnout. Negative emotional experiences were more common than positive ones and were more strongly correlated with burnout. Other studies of mothers have similarly found that social media groups can evoke various negative emotions, despite connective benefits (César et al., 2018). In addition, in our study the impact of time spent on social media was less than that of either positive or negative emotions felt when comparing oneself to others on social media.

Thus, emotional reactions when using social media at least partially determine its effects. Social media use may contribute to burnout because women more often experience negative instead of positive emotions, when comparing themselves to others. These findings are both disconcerting, given the high use of social media in our current culture, and potentially promising, in that they highlight avenues for interventions to limit social comparisons *via* social media that are likely to trigger negative emotional responses (e.g., *via* cognitive, emotional, and/or social reframing techniques), or to facilitate more realistic and positive social comparisons. In interpreting our findings, we note that we assessed four emotions in response to social media, but additional emotions could be assessed (e.g., Park and Baek, 2018).

Complementing our findings on the impacts of social comparisons in general and on social media, a do it all discrepancy was also linked to burnout. This is important to note, given the often-prevailing message that women today can “do and have it all” and the expectations placed on women in intensive mothering cultures (Lamar et al., 2019). Our findings suggest that women who feel a large gap between what they should do and what they can do are at a higher risk of burnout and may be more impacted by social comparisons. In contrast, a near-zero or even negative do it all discrepancy may be protective against burnout and the negative impacts of social comparisons. These benefits may be linked to a stronger sense of self-efficacy (e.g., Bandura, 2006), additional internal or external resources, or simply more realistic should-do expectations.

While social support, particularly from the non-work domain, appears connected to lower levels of work-related and parental burnout in line with prior research (e.g., Ardic, 2020; Szczygiel et al., 2020; Velando-Soriano et al., 2020), it does not seem to alter the impacts of social media or social comparisons on burnout. The one exception to this pattern was our finding that work social support appears most influential in weakening negative impacts of social comparisons when individuals already have a lower do it all discrepancy. These findings initially were somewhat surprising, given the large literature showing the benefits of social support on health and wellbeing. Still, support not sufficiently matched to a demand may be minimally helpful (Beehr et al., 2010). In addition, the demands-control-support framework (Van Der Doef and Maes, 1999) also emphasizes the stronger power of support when combined with high perceived control, which is often operationalized as self-efficacy or a strong can-do perception. Our findings support the notion that the availability of support may not enough to offset the cognitive/emotional demands of more internal comparisons to others and personal expectations.

### Limitations

As with any research, the present study has limitations. First, our sample was relatively resource-rich in terms of education, income, and availability of personal relationships. Our findings may not generalize fully to other populations. Future studies can establish whether the effects we observed are even more pronounced in less resource-rich populations, especially single mothers and/or minority women who may experience heightened comparison concerns related to stereotypes. Indeed, there is a need for strategies to better reach such populations, given the large gap in our understanding of burnout within minority populations.

Second, all data were gathered *via* self-report measures. This was necessary given the perceptual nature of our constructs. However, future studies could incorporate additional, more objective indicators such as software-monitored social media usage for more accurate time assessments and social network communication patterns, and other indicators of health and wellbeing.

Third, our mediation tests were conducted with cross-sectional data. We carefully ensured the ordering of our variables was theoretically and logically sound (i.e., that time spent on social media would reasonably lead to emotions experienced when on social media). Still, future research is encouraged to gather longitudinal data from respondents, separating antecedents, intervening variables, and outcomes, with careful consideration to appropriate timeframes for these effects to unfold.

### Practical implications and future directions

Our findings support multiple practice and research implications. First, our study illustrates the value of considering burnout in multiple life domains. Parental burnout and work-related burnout were moderately correlated, suggesting that burnout is not experienced or limited in its effects to one domain. Still, we found some nuances in the social forces that relate to burnout in each domain. Second, these data highlight a need – and potentially broad beneficial impact - for interventions to facilitate more positive emotions and more accurate and positive social comparisons when using social media. Interventions could target individual users (e.g., recognize and avoid accounts or posts that trigger negative emotions or self-comparisons; structure feeds to encourage positive emotions; learn that what we see on social media is not always reality), but could also target the ways in which social media groups facilitate interactions (e.g., discussion forums around realistic expectations; rules of engagement for positive interactions). We also encourage a reassessment of the language and perception of “do it all” that remains popular, and a better understanding of how to set and maintain realistic expectations for oneself and others. Intentional discussions can facilitate acknowledgment and authentic demonstration of our personal limits and that it is ok to *not* do it all, as well as build efficacy for what one can do.

Third, the lack of support for our anticipated interaction effects highlights the need for social support to match the situation to be helpful. From our research, “the situation” includes not only the work or family role domain, but also the individual’s personal state of mind and overall set of available resources. Theories may need to consider possibility that social media use may constitute its own role domain, akin to well-studied work or family domains but with its own parameters that must be better understood. It may also be that individual differences related to susceptibility for making or being affected by social comparisons are more critical moderators for future research. Further, studies could look at a greater variety of emotional reactions to social media as contributing factors. We encourage further study into why some people are lifted up versus let down by social media engagements.

It is our hope that this study, and future studies, can begin to change the impact of social forces around us to foster positive emotional experiences, where our social context protects us from burnout, rather than puts us at risk.

## Data availability statement

The raw data supporting the conclusions of this article will be made available by the authors, without undue reservation.

## Ethics statement

The studies involving human participants were reviewed and approved by the University of Tennessee at Chattanooga Institutional Review Board. The participants provided their written informed consent to participate in this study.

## Author contributions

KB carried out analyses and led the writing of the sections “Introduction” and “Results.” CC carried out data processing and data cleaning and led the writing of the sections “Methods” and “Discussion.” DG led efforts to recruit participants and assisted in writing the sections “Introduction” and “Discussion.” KW assisted with logistics planning throughout study development and data collection and assisted in writing the section “Introduction.” All authors meaningfully contributed to the development and implementation of the survey and study design, as well as the writing and reviewing of the manuscript.
