# Supplementary material for: Understudied social influences on work-related and parental burnout: Social media-related emotions, comparisons, and the “do it all discrepancy”
Source: Front Psychol. 2022 Sep 21;13:977782. doi: 10.3389/fpsyg.2022.977782 (PMC9532694; doi:10.3389/fpsyg.2022.977782)
Supplement: Supplementary file 1 [file Data_Sheet_1.docx]

**Supplemental Figure 1.**

*Effect of social comparisons on parental burnout, moderated by do it all discrepancy.*
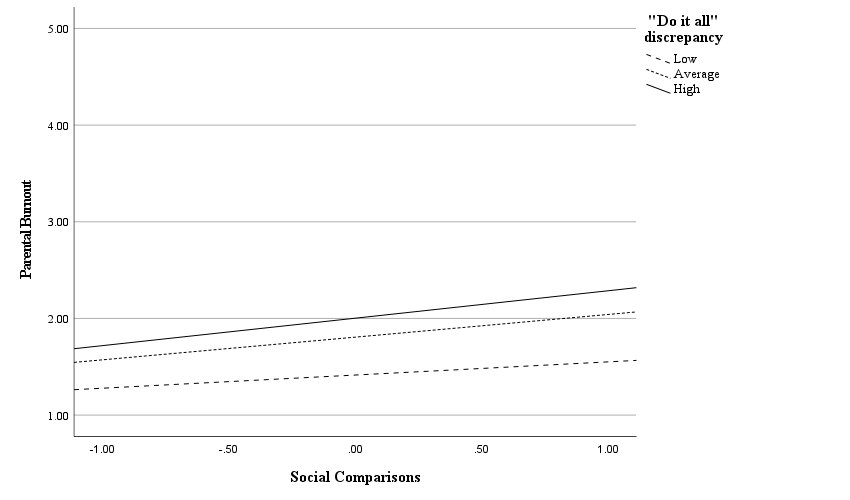


**Supplemental Figure 2.**

*Effect of social comparisons on work-related burnout, moderated by do it all discrepancy and work support*


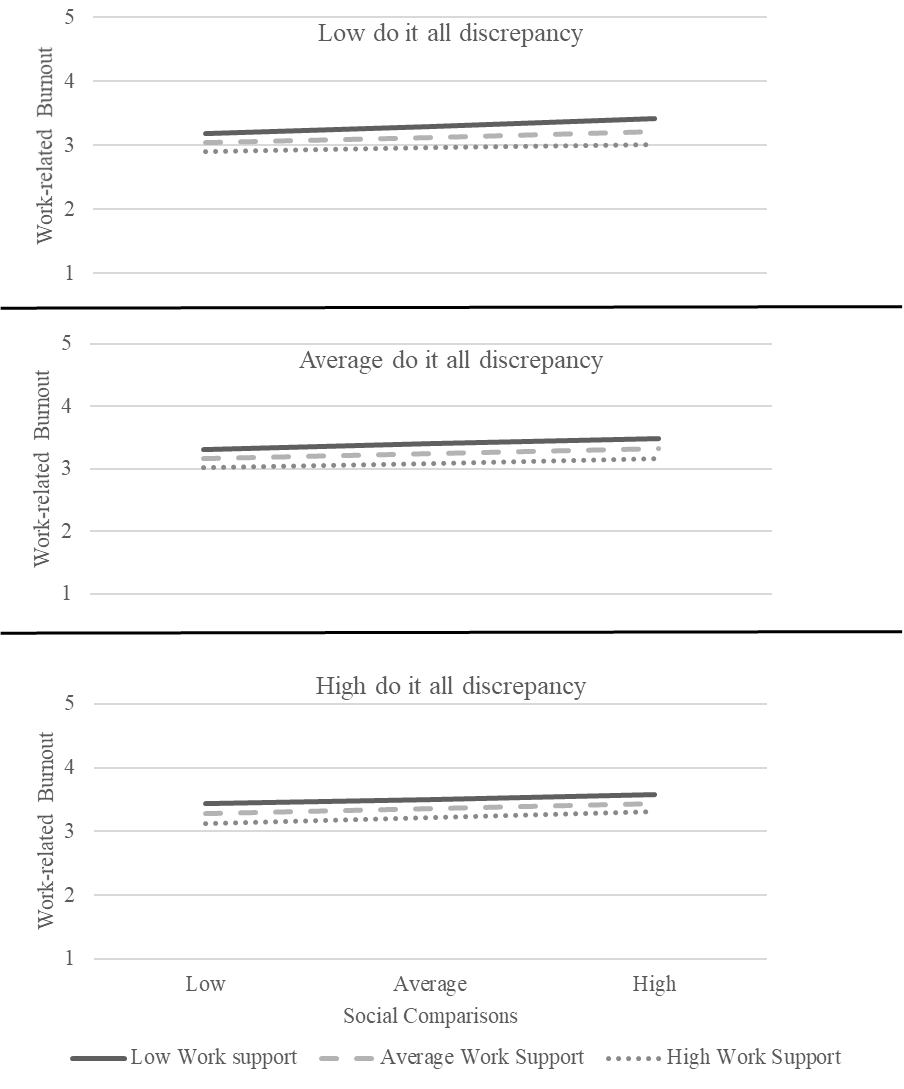


**Supplemental Table 1.** Moderation and mediation tests for Hypothesis 1.

|  | **Work-Related Burnout Models** | | | | | |  | **Parental Burnout Models** | | | | | |
| --- | --- | --- | --- | --- | --- | --- | --- | --- | --- | --- | --- | --- | --- |
|  | ***Model 1.1:*** | | | | | |  | ***Model 1.2:*** | | | | | |
|  | ***Outcome: Negative emotions social media (R^2^ = .11)*** | | | | | |  | ***Outcome: Negative emotions social media (R^2^ = .07)*** | | | | | |
|  | *b* | *se* | *t* | *p* | CI Lower | CI Upper |  | *b* | *se* | *t* | *p* | CI Lower | CI Upper |
| Constant | 0.42 | 0.17 | 2.43 | 0.02 | 0.082 | 0.767 |  | 0.31 | 0.20 | 1.57 | 0.12 | -0.077 | 0.704 |
| Age | -0.02 | 0.00 | -11.85 | 0.00 | -0.027 | -0.019 |  | -0.02 | 0.00 | -6.97 | 0.00 | -0.024 | -0.014 |
| Education | 0.02 | 0.01 | 1.23 | 0.22 | -0.009 | 0.039 |  | 0.02 | 0.01 | 1.54 | 0.12 | -0.006 | 0.048 |
| Marital Status | -0.07 | 0.05 | -1.35 | 0.18 | -0.179 | 0.033 |  | -0.21 | 0.08 | -2.64 | 0.01 | -0.361 | -0.053 |
| Number Dependents | 0.00 | 0.02 | -0.07 | 0.95 | -0.034 | 0.032 |  | -0.01 | 0.02 | -0.52 | 0.60 | -0.054 | 0.031 |
| Work Hours | -0.06 | 0.04 | -1.66 | 0.10 | -0.129 | 0.011 |  | -0.09 | 0.04 | -2.26 | 0.02 | -0.163 | -0.012 |
| Volunteer Hours | -0.01 | 0.01 | -0.60 | 0.55 | -0.037 | 0.020 |  | -0.02 | 0.02 | -1.37 | 0.17 | -0.054 | 0.010 |
| Home Overload | 0.10 | 0.02 | 4.62 | 0.00 | 0.058 | 0.143 |  | 0.12 | 0.02 | 4.89 | 0.00 | 0.072 | 0.169 |
| Work Overload | 0.04 | 0.02 | 2.88 | 0.00 | 0.014 | 0.075 |  | 0.02 | 0.02 | 1.31 | 0.19 | -0.011 | 0.056 |
| SM Hours | 0.01 | 0.00 | 4.56 | 0.00 | 0.007 | 0.017 |  | 0.01 | 0.00 | 3.43 | 0.00 | 0.004 | 0.016 |
| Nonwork Support (NWS) | -0.17 | 0.03 | -6.88 | 0.00 | -0.222 | -0.123 |  | -0.15 | 0.03 | -5.48 | 0.00 | -0.205 | -0.097 |
| SM Hours * NWS | 0.00 | 0.00 | -0.95 | 0.34 | -0.011 | 0.004 |  | 0.00 | 0.00 | -0.98 | 0.33 | -0.012 | 0.004 |
| Work Support (WS) | -0.01 | 0.02 | -0.56 | 0.58 | -0.045 | 0.025 |  | -0.02 | 0.02 | -0.88 | 0.38 | -0.056 | 0.021 |
| SM Hours * WS | 0.00 | 0.00 | 0.24 | 0.81 | -0.005 | 0.006 |  | 0.00 | 0.00 | 0.45 | 0.65 | -0.004 | 0.007 |
|  | ***Outcome: Work-related burnout (R^2^ = .31)*** | | | | | |  | ***Outcome: Parental burnout (R^2^ = .21)*** | | | | | |
| Constant | 2.17 | 0.11 | 19.73 | 0.00 | 1.957 | 2.389 |  | 2.18 | 0.27 | 8.06 | 0.00 | 1.648 | 2.708 |
| Age | -0.01 | 0.00 | -6.27 | 0.00 | -0.010 | -0.005 |  | -0.04 | 0.00 | -9.43 | 0.00 | -0.042 | -0.028 |
| Education | 0.00 | 0.01 | -0.31 | 0.75 | -0.018 | 0.013 |  | 0.04 | 0.02 | 2.28 | 0.02 | 0.006 | 0.079 |
| Marital Status | 0.04 | 0.03 | 1.27 | 0.20 | -0.024 | 0.110 |  | 0.00 | 0.11 | 0.04 | 0.97 | -0.205 | 0.212 |
| Number Dependents | 0.01 | 0.01 | 0.87 | 0.38 | -0.012 | 0.030 |  | 0.11 | 0.03 | 3.83 | 0.00 | 0.055 | 0.171 |
| Work Hours | 0.07 | 0.02 | 3.22 | 0.00 | 0.028 | 0.116 |  | -0.26 | 0.05 | -4.92 | 0.00 | -0.361 | -0.155 |
| Volunteer Hours | -0.02 | 0.01 | -1.77 | 0.08 | -0.034 | 0.002 |  | 0.00 | 0.02 | 0.03 | 0.98 | -0.043 | 0.044 |
|  |  |  |  |  |  |  |  |  |  |  |  |  |  |
|  | *b* | *se* | *t* | *p* | CI Lower | CI Upper |  | *b* | *se* | *t* | *p* | CI Lower | CI Upper |
| Home Overload | 0.15 | 0.01 | 11.10 | 0.00 | 0.125 | 0.179 |  | 0.24 | 0.03 | 7.19 | 0.00 | 0.176 | 0.308 |
| Work Overload | 0.14 | 0.01 | 13.78 | 0.00 | 0.116 | 0.154 |  | 0.05 | 0.02 | 1.95 | 0.05 | 0.000 | 0.092 |
| SM Hours | 0.00 | 0.00 | 2.66 | 0.01 | 0.001 | 0.008 |  | 0.01 | 0.00 | 2.03 | 0.04 | 0.000 | 0.016 |
| SM Negative Emotions (SMNE) | 0.09 | 0.01 | 8.06 | 0.00 | 0.071 | 0.117 |  | 0.24 | 0.03 | 8.75 | 0.00 | 0.190 | 0.299 |
| Nonwork Support (NWS) | -0.13 | 0.02 | -8.22 | 0.00 | -0.162 | -0.100 |  | -0.49 | 0.04 | -12.99 | 0.00 | -0.562 | -0.415 |
| SM Hours * NWS | 0.00 | 0.00 | -1.18 | 0.24 | -0.007 | 0.002 |  | 0.01 | 0.01 | 1.31 | 0.19 | -0.004 | 0.018 |
| SM NE * NWS | 0.02 | 0.02 | 1.47 | 0.14 | -0.008 | 0.055 |  | -0.01 | 0.04 | -0.30 | 0.77 | -0.089 | 0.066 |
| Work Support (WS) | -0.14 | 0.01 | -12.62 | 0.00 | -0.165 | -0.121 |  | -0.02 | 0.03 | -0.77 | 0.44 | -0.074 | 0.032 |
| SM Hours * WS | 0.00 | 0.00 | 0.95 | 0.34 | -0.002 | 0.005 |  | -0.01 | 0.00 | -1.41 | 0.16 | -0.013 | 0.002 |
| SM NE * WS | -0.01 | 0.01 | -1.06 | 0.29 | -0.035 | 0.011 |  | -0.05 | 0.03 | -1.55 | 0.12 | -0.104 | 0.012 |
|  |  |  |  |  |  |  |  | |  |  |  |  |  |
|  | NWS | WS | Effect | BootSE | CI Lower | CI Upper |  | NWS | WS | Effect | BootSE | CI Lower | CI Upper |
| ***Bootstrapped Confidence Intervals for Indirect Effects via Negative Emotions*** | -1 SD | -1 SD | 0.0012 | 0.0004 | **0.0005** | **0.0022** |  | -1 SD | -1 SD | 0.0034 | 0.0013 | **0.0010** | **0.0062** |
|  | -1 SD | Avg | 0.0011 | 0.0004 | **0.0005** | **0.0019** |  | -1 SD | Avg | 0.0032 | 0.0011 | **0.0013** | **0.0056** |
|  | -1 SD | +1 SD | 0.0010 | 0.0005 | **0.0002** | **0.0021** |  | -1 SD | +1 SD | 0.0029 | 0.0014 | **0.0007** | **0.0060** |
|  | Avg | -1 SD | 0.0012 | 0.0004 | **0.0004** | **0.0021** |  | Avg | -1 SD | 0.0025 | 0.0012 | **0.0002** | **0.0048** |
|  | Avg | Avg | 0.0011 | 0.0003 | **0.0006** | **0.0017** |  | Avg | Avg | 0.0024 | 0.0008 | **0.0009** | **0.0041** |
|  | Avg | +1 SD | 0.0010 | 0.0004 | **0.0004** | **0.0018** |  | Avg | +1 SD | 0.0023 | 0.0010 | **0.0006** | **0.0044** |
|  | +1 SD | -1 SD | 0.0011 | 0.0007 | -0.0002 | 0.0024 |  | +1 SD | -1 SD | 0.0017 | 0.0016 | -0.0017 | 0.0047 |
|  | +1 SD | Avg | 0.0010 | 0.0005 | **0.0002** | **0.0020** |  | +1 SD | Avg | 0.0017 | 0.0011 | -0.0004 | 0.0039 |
|  | +1 SD | +1 SD | 0.0010 | 0.0005 | **0.0002** | **0.0020** |  | +1 SD | +1 SD | 0.0016 | 0.0010 | -0.0002 | 0.0038 |
|  |  |  |  |  |  |  |  |  |  |  |  |  |  |
|  | ***Model 1.3:*** | | | | | |  | ***Model 1.4:*** | | | | | |
|  | ***Outcome: Positive emotions social media (R^2^ = .05)*** | | | | | |  | ***Outcome: Positive emotions social media (R^2^ = .05)*** | | | | | |
|  | *b* | *se* | *t* | *p* | CI Lower | CI Upper |  | *b* | *se* | *t* | *p* | CI Lower | CI Upper |
| Constant | -0.08 | 0.16 | -0.51 | 0.61 | -0.401 | 0.235 |  | 0.11 | 0.18 | 0.62 | 0.54 | -0.247 | 0.474 |
| Age | 0.00 | 0.00 | 1.91 | 0.06 | 0.000 | 0.007 |  | 0.00 | 0.00 | 0.13 | 0.90 | -0.005 | 0.005 |
| Education | -0.02 | 0.01 | -1.63 | 0.10 | -0.041 | 0.004 |  | -0.03 | 0.01 | -2.21 | 0.03 | -0.053 | -0.003 |
| Marital Status | -0.06 | 0.05 | -1.21 | 0.23 | -0.159 | 0.038 |  | -0.01 | 0.07 | -0.18 | 0.86 | -0.155 | 0.129 |
| Number Dependents | 0.03 | 0.02 | 2.01 | 0.05 | 0.001 | 0.062 |  | 0.03 | 0.02 | 1.28 | 0.20 | -0.014 | 0.065 |
| Work Hours | 0.03 | 0.03 | 0.94 | 0.35 | -0.034 | 0.096 |  | 0.03 | 0.04 | 0.74 | 0.46 | -0.043 | 0.096 |
| Volunteer Hours | 0.03 | 0.01 | 2.38 | 0.02 | 0.006 | 0.058 |  | 0.03 | 0.02 | 1.87 | 0.06 | -0.001 | 0.058 |
| Home Overload | -0.06 | 0.02 | -2.99 | 0.00 | -0.100 | -0.021 |  | -0.06 | 0.02 | -2.83 | 0.00 | -0.109 | -0.020 |
| Work Overload | 0.03 | 0.01 | 2.17 | 0.03 | 0.003 | 0.060 |  | 0.04 | 0.02 | 2.23 | 0.03 | 0.004 | 0.067 |
| SM Hours | 0.02 | 0.00 | 8.38 | 0.00 | 0.016 | 0.025 |  | 0.02 | 0.00 | 7.56 | 0.00 | 0.015 | 0.026 |
| Nonwork Support (NWS) | 0.08 | 0.02 | 3.39 | 0.00 | 0.033 | 0.124 |  | 0.08 | 0.03 | 3.27 | 0.00 | 0.033 | 0.133 |
| SM Hours * NWS | 0.00 | 0.00 | -0.22 | 0.82 | -0.008 | 0.006 |  | 0.00 | 0.00 | -0.52 | 0.60 | -0.009 | 0.006 |
| Work Support (WS) | 0.09 | 0.02 | 5.14 | 0.00 | 0.053 | 0.118 |  | 0.09 | 0.02 | 4.84 | 0.00 | 0.053 | 0.124 |
| SM Hours * WS | 0.00 | 0.00 | -0.93 | 0.35 | -0.007 | 0.003 |  | 0.00 | 0.00 | -0.67 | 0.51 | -0.007 | 0.003 |
|  | ***Outcome: Work-related burnout (R^2^ = .30)*** | | | | | |  | ***Outcome: parental burnout (R^2^ = .20)*** | | | | | |
| Constant | 2.20 | 0.11 | 19.86 | 0.00 | 1.987 | 2.422 |  | 2.25 | 0.27 | 8.21 | 0.00 | 1.711 | 2.784 |
| Age | -0.01 | 0.00 | -7.98 | 0.00 | -0.012 | -0.007 |  | -0.04 | 0.00 | -10.56 | 0.00 | -0.047 | -0.032 |
| Education | 0.00 | 0.01 | -0.26 | 0.79 | -0.017 | 0.013 |  | 0.04 | 0.02 | 2.33 | 0.02 | 0.007 | 0.081 |
| Marital Status | 0.03 | 0.03 | 0.94 | 0.35 | -0.035 | 0.099 |  | -0.05 | 0.11 | -0.43 | 0.67 | -0.257 | 0.165 |
| Number Dependents | 0.01 | 0.01 | 1.06 | 0.29 | -0.010 | 0.032 |  | 0.12 | 0.03 | 3.88 | 0.00 | 0.057 | 0.174 |
| Work Hours | 0.07 | 0.02 | 3.05 | 0.00 | 0.025 | 0.113 |  | -0.27 | 0.05 | -5.11 | 0.00 | -0.376 | -0.167 |
| Volunteer Hours | -0.01 | 0.01 | -1.56 | 0.12 | -0.032 | 0.004 |  | 0.00 | 0.02 | 0.00 | 1.00 | -0.044 | 0.044 |
| Home Overload | 0.16 | 0.01 | 11.46 | 0.00 | 0.131 | 0.185 |  | 0.26 | 0.03 | 7.64 | 0.00 | 0.192 | 0.325 |
| Work Overload | 0.14 | 0.01 | 14.35 | 0.00 | 0.122 | 0.161 |  | 0.06 | 0.02 | 2.47 | 0.01 | 0.012 | 0.105 |
| SM Hours | 0.01 | 0.00 | 4.03 | 0.00 | 0.004 | 0.010 |  | 0.01 | 0.00 | 3.40 | 0.00 | 0.006 | 0.022 |
|  | *b* | *se* | *t* | *p* | CI Lower | CI Upper |  | *b* | *se* | *t* | *p* | CI Lower | CI Upper |
| SM Positive Emotions (SMPE) | -0.06 | 0.01 | -4.98 | 0.00 | -0.088 | -0.039 |  | -0.15 | 0.03 | -4.95 | 0.00 | -0.212 | -0.092 |
| Nonwork Support (NWS) | -0.14 | 0.02 | -8.91 | 0.00 | -0.173 | -0.111 |  | -0.51 | 0.04 | -13.42 | 0.00 | -0.582 | -0.434 |
| SM Hours * NWS | 0.00 | 0.00 | -1.14 | 0.25 | -0.008 | 0.002 |  | 0.00 | 0.01 | 0.81 | 0.42 | -0.007 | 0.016 |
| SM PE * NWS | 0.00 | 0.02 | -0.20 | 0.85 | -0.042 | 0.034 |  | 0.06 | 0.05 | 1.31 | 0.19 | -0.031 | 0.153 |
| Work Support (WS) | -0.14 | 0.01 | -12.09 | 0.00 | -0.161 | -0.116 |  | -0.01 | 0.03 | -0.37 | 0.71 | -0.064 | 0.043 |
| SM Hours * WS | 0.00 | 0.00 | 0.75 | 0.45 | -0.002 | 0.005 |  | -0.01 | 0.00 | -1.39 | 0.17 | -0.013 | 0.002 |
| SM PE * WS | 0.00 | 0.01 | 0.03 | 0.98 | -0.026 | 0.027 |  | -0.01 | 0.03 | -0.42 | 0.68 | -0.080 | 0.052 |
|  |  |  |  |  |  |  |  | |  |  |  |  |  |
|  | NWS | WS | Effect | BootSE | CI Lower | CI Upper |  | NWS | WS | Effect | BootSE | CI Lower | CI Upper |
| ***Bootstrapped Confidence Intervals for Indirect Effects via Positive Emotions*** | -1 SD | -1 SD | -0.0014 | 0.0006 | **-0.0026** | **-0.0004** |  | -1 SD | -1 SD | -0.0042 | 0.0015 | **-0.0076** | **-0.0015** |
|  | -1 SD | Avg | -0.0013 | 0.0005 | **-0.0023** | **-0.0004** |  | -1 SD | Avg | -0.0042 | 0.0013 | **-0.0070** | **-0.0020** |
|  | -1 SD | +1 SD | -0.0011 | 0.0006 | **-0.0024** | **-0.0001** |  | -1 SD | +1 SD | -0.0041 | 0.0016 | **-0.0076** | **-0.0014** |
|  | Avg | -1 SD | -0.0014 | 0.0005 | **-0.0025** | **-0.0005** |  | Avg | -1 SD | -0.0030 | 0.0012 | **-0.0056** | **-0.0009** |
|  | Avg | Avg | -0.0013 | 0.0003 | **-0.0020** | **-0.0007** |  | Avg | Avg | -0.0031 | 0.0008 | **-0.0047** | **-0.0017** |
|  | Avg | +1 SD | -0.0011 | 0.0004 | **-0.0021** | **-0.0004** |  | Avg | +1 SD | -0.0031 | 0.0010 | **-0.0053** | **-0.0013** |
|  | +1 SD | -1 SD | -0.0015 | 0.0007 | **-0.0030** | **-0.0002** |  | +1 SD | -1 SD | -0.0020 | 0.0014 | -0.0050 | 0.0004 |
|  | +1 SD | Avg | -0.0013 | 0.0005 | **-0.0023** | **-0.0005** |  | +1 SD | Avg | -0.0021 | 0.0009 | **-0.0042** | **-0.0005** |
|  | +1 SD | +1 SD | -0.0012 | 0.0005 | **-0.0021** | **-0.0004** |  | +1 SD | +1 SD | -0.0021 | 0.0010 | **-0.0043** | **-0.0004** |
| Notes. N = 2904 for work-related burnout models; N = 2376 for parental burnout models. SM = Social media. Education coded as highest degree earned, higher values represent a more advanced degree. Martial status coded 0 = married or committed relationship, 1 = single, widowed, or divorced. Bolded confidence intervals indicate the significance of the indirect effects. | | | | | | | | | | | | | |

**Supplemental Table 2.** Moderated regression analyses testing Hypothesis 2.

|  | **Work-Related Burnout** | | | | | |  | **Parental Burnout** | | | | | | | |
| --- | --- | --- | --- | --- | --- | --- | --- | --- | --- | --- | --- | --- | --- | --- | --- |
|  | ***Model 2.1: Nonwork support as moderator (Model R^2^ = .29)*** | | | | | |  | ***Model 2.2: Nonwork support as moderator (Model R^2^ = .24)*** | | | | | | | |
|  | *b* | *SE* | *t* | *p* | Lower CI | Upper CI |  | *b* | *SE* | *t* | *p* | Lower CI | Upper CI | | |
| Constant | 2.47 | 0.12 | 21.28 | <.001 | 2.24 | 2.69 |  | 2.52 | 0.26 | 9.73 | 0.00 | 2.01 | 3.03 | | |
| Age | -0.01 | 0.00 | -4.89 | <.001 | -0.01 | 0.00 |  | -0.03 | 0.00 | -9.25 | 0.00 | -0.04 | -0.03 | | |
| Education | -0.01 | 0.01 | -0.72 | 0.47 | -0.02 | 0.01 |  | 0.04 | 0.02 | 2.47 | 0.01 | 0.01 | 0.08 | | |
| Marital Status | 0.07 | 0.04 | 1.62 | 0.10 | -0.01 | 0.15 |  | 0.01 | 0.10 | 0.08 | 0.94 | -0.19 | 0.21 | | |
| Number of Dependents | 0.01 | 0.01 | 0.90 | 0.37 | -0.01 | 0.03 |  | 0.13 | 0.03 | 4.48 | 0.00 | 0.07 | 0.18 | | |
| Work Hours | 0.00 | 0.02 | 0.16 | 0.87 | -0.04 | 0.04 |  | -0.31 | 0.05 | -6.57 | 0.00 | -0.40 | -0.22 | | |
| Volunteer Hours | -0.01 | 0.01 | -1.38 | 0.17 | -0.03 | 0.01 |  | -0.01 | 0.02 | -0.27 | 0.79 | -0.05 | 0.04 | | |
| Home Overload | 0.13 | 0.01 | 8.54 | <.001 | 0.10 | 0.16 |  | 0.19 | 0.03 | 5.54 | 0.00 | 0.12 | 0.25 | | |
| Work Overload | 0.13 | 0.01 | 12.68 | <.001 | 0.11 | 0.15 |  | 0.04 | 0.02 | 1.60 | 0.11 | -0.01 | 0.08 | | |
| Social Comparisons (SC) | 0.09 | 0.01 | 6.65 | <.001 | 0.06 | 0.11 |  | 0.19 | 0.03 | 6.53 | 0.00 | 0.14 | 0.25 | | |
| Non-Work Support (NWS) | -0.18 | 0.02 | -10.77 | <.001 | -0.21 | -0.15 |  | -0.47 | 0.04 | -13.03 | 0.00 | -0.55 | -0.40 | | |
| Do it all discrepancy (DD) | 0.08 | 0.01 | 9.78 | <.001 | 0.06 | 0.10 |  | 0.18 | 0.02 | 9.97 | 0.00 | 0.14 | 0.22 | | |
| SC*NWS | 0.01 | 0.02 | 0.58 | 0.56 | -0.03 | 0.05 |  | -0.01 | 0.04 | -0.34 | 0.73 | -0.10 | 0.07 | | |
| SC*DD | 0.00 | 0.01 | 0.39 | 0.70 | -0.01 | 0.02 |  | 0.05 | 0.02 | 2.67 | 0.01 | 0.01 | 0.09 | | |
| NWS*DD | 0.02 | 0.01 | 1.39 | 0.17 | -0.01 | 0.04 |  | 0.01 | 0.03 | 0.42 | 0.67 | -0.04 | 0.06 | | |
| SC*NWS*DD | 0.02 | 0.01 | 1.38 | 0.17 | -0.01 | 0.04 |  | 0.03 | 0.03 | 1.14 | 0.25 | -0.02 | 0.08 | | |
|  |  |  |  |  |  |  |  | |  |  |  |  |  |  |  |
|  | ***Model 2.3: Work support as moderator (Model R^2^ = .32)*** | | | | | |  | ***Model 2.4: Work support as moderator (Model R^2^ = .19)*** | | | | | | |  |
|  | *b* | *SE* | *t* | *p* | Lower CI | Upper CI |  | *b* | *SE* | *t* | *p* | Lower CI | Upper CI | | |
| Constant | 2.32 | 0.12 | 19.71 | <.001 | 2.09 | 2.55 |  | 2.16 | 0.28 | 7.83 | 0.00 | 1.62 | 2.70 | | |
| Age | -0.01 | 0.00 | -4.96 | <.001 | -0.01 | 0.00 |  | -0.03 | 0.00 | -7.94 | 0.00 | -0.04 | -0.02 | | |
| Education | -0.01 | 0.01 | -0.81 | 0.42 | -0.02 | 0.01 |  | 0.02 | 0.02 | 1.29 | 0.20 | -0.01 | 0.06 | | |
| Marital Status | 0.10 | 0.04 | 2.33 | 0.02 | 0.02 | 0.18 |  | 0.16 | 0.11 | 1.50 | 0.13 | -0.05 | 0.37 | | |
| Number of Dependents | 0.02 | 0.01 | 1.82 | 0.07 | 0.00 | 0.04 |  | 0.14 | 0.03 | 4.70 | 0.00 | 0.08 | 0.20 | | |
| Work Hours | 0.04 | 0.02 | 1.92 | 0.05 | 0.00 | 0.09 |  | -0.27 | 0.05 | -5.13 | 0.00 | -0.38 | -0.17 | | |
| Volunteer Hours | -0.01 | 0.01 | -1.38 | 0.17 | -0.03 | 0.01 |  | -0.01 | 0.02 | -0.45 | 0.65 | -0.05 | 0.03 | | |
| Home Overload | 0.14 | 0.01 | 9.29 | <.001 | 0.11 | 0.17 |  | 0.23 | 0.03 | 6.60 | 0.00 | 0.16 | 0.30 | | |
| Work Overload | 0.13 | 0.01 | 12.25 | <.001 | 0.11 | 0.15 |  | 0.04 | 0.02 | 1.53 | 0.13 | -0.01 | 0.08 | | |
| Social Comparisons (SC) | 0.10 | 0.01 | 7.42 | <.001 | 0.07 | 0.12 |  | 0.23 | 0.03 | 7.52 | 0.00 | 0.17 | 0.29 | | |
| Work Support (WS) | -0.17 | 0.01 | -14.23 | <.001 | -0.19 | -0.14 |  | -0.10 | 0.03 | -3.75 | 0.00 | -0.15 | -0.05 | | |
| Do it all discrepancy (DD) | 0.08 | 0.01 | 10.14 | <.001 | 0.07 | 0.10 |  | 0.19 | 0.02 | 10.41 | 0.00 | 0.16 | 0.23 | | |
| SC*WS | -0.01 | 0.01 | -0.82 | 0.41 | -0.04 | 0.01 |  | -0.05 | 0.03 | -1.62 | 0.11 | -0.11 | 0.01 | | |
| SC*DD | 0.00 | 0.01 | -0.07 | 0.95 | -0.02 | 0.02 |  | 0.04 | 0.02 | 2.14 | 0.03 | 0.00 | 0.08 | | |
| WS*DD | 0.01 | 0.01 | 1.13 | 0.26 | -0.01 | 0.03 |  | 0.03 | 0.02 | 1.39 | 0.17 | -0.01 | 0.06 | | |
| SC*WS*DD | 0.02 | 0.01 | 2.69 | 0.01 | 0.01 | 0.04 |  | 0.03 | 0.02 | 1.38 | 0.17 | -0.01 | 0.06 | | |

*Notes*. Model 2.1 *N =* 2633. Model 2.2 *N =* 2443. Model 2.3 *N =* 2569. Model 2.4 *N* = 2389. Education coded as highest degree earned, higher values represent a more advanced degree. Martial status: 0 = married or committed relationship, 1 = single, widowed, or divorced.
